# Evaluation of Failure Patterns Using Trimodality in Non-Small Cell Lung Cancer

**DOI:** 10.4021/wjon289w

**Published:** 2011-04-09

**Authors:** Shilpen Patel, Janelle Pakish, Philemon Yen, Tony Quang, Laurie Carr, Douglas Wood, Keith Eaton, Michael Mulligan, Renato Martins

**Affiliations:** a Department of Radiation Oncology, University of Washington Medical Center, Seattle, WA, USA; b Puget Sound Veterans Administration Seattle, WA, USA; c Department of Medical Oncology, Seattle Cancer Care Alliance, Seattle, WA, USA; d Department of Thoracic Surgery, University of Washington Medical Center, Seattle, WA, USA

**Keywords:** Non-small cell lung cancer, Trimodality, Failure patterns

## Abstract

**Background:**

The effectiveness of trimodality therapy in NSCLC has been controversial.

**Methods:**

Ninety-two patients with stage III NSCLC were analyzed retrospectively based on treatment given. Overall survival (OS) and patterns of failure were examined in patients treated with chemoradiation alone (Group 1) versus neoadjuvant chemoradiation followed by surgical resection (Group 2).

**Results:**

OS for 2, 3, and 5 years in Group 1 and 2 were 19.7%, 15.7%, and 4.5% versus 56.4%, 40.4%, and 32.3% (P = 0.003), respectively. Median survival for Group 1 and 2 was 11.0 and 34.0 months, respectively (P = 0.003). The recurrence rate in Group 1 was 61.8% (47 of 76) with distant non-brain involvement (48.9%). In Group 2 it was 50.0% (8 of 16) with brain (50%) involvement.

**Conclusions:**

Patients with stage IIIA and, perhaps IIIB NSCLC with a high performance status should be considered for trimodality treatment.

## Introduction

Despite the development of new treatments in oncology, lung cancer remains the leading cause of cancer mortality among both men and women. Both short and long term survival have improved, but the optimal management of this disease still remains unresolved. The current standard of care in this country for locally advanced non-small cell lung cancer (NSCLC) proposes a bimodality approach of concurrent chemotherapy and radiation therapy [1-3]. Studies performed by the Southwest Oncology Group (SWOG) and the Radiation Therapy Oncology Group (RTOG) have found that patients with stage IIIB can be definitively treated with concurrent chemoradiation, including a radiation dose of 61 - 63 Gy, safely with increased survival [1, 2]. Compared to 3 year overall survival (OS) of patients treated with radiation alone or sequential chemotherapy followed by radiation these studies showed an improvement of 17 - 28% and versus less than 10% [1, 2, 4].

Additionally, a literature review of prospective randomized trials and large phase II studies by Edelman et al evaluated several studies comparing outcomes of concurrent chemoradiation to radiation therapy alone. They found 2 year OS to range from 19 - 40% in the concurrent chemoradiation groups versus 9 - 22% with radiation therapy alone [3]. Although these studies demonstrate improvement from single modality therapies, the improvement is small and not favorable as a goal for long term survival in this commonly progressive cancer.

Furthermore, despite new developments in each of these therapies, both local and distant control continues to remain a problem [1]. The Southwest Group study showed increased OS but found local disease progression in 23% and distant metastasis in 65% of patients. These high rates of recurrence are unfavorable and thus create a focus and need for future investigation.

Previous studies have investigated the effectiveness of trimodality therapy of chemotherapy, radiotherapy, and surgery in stage IIIA and IIIB NSCLC. The results have been inconclusive and no new standard of care has been defined [5-11]. The initial results from the phase III NSCLC Intergroup 0139 study showed a prolonged disease free progression compared to concurrent chemoradiation (14.0 v 11.7 months), but there was no difference found in OS (22 months) [6]. An Italian study showed a promising 5 year OS of 38.0% in trimodality group compared to 5.6% in the concurrent chemoradiation group [9]. This was a small study, however, with only seven patients [9]. Lastly, a retrospective study by Sonett et al investigated the possibility of treating patients with induction concurrent chemoradiation that included definitive radiation dose up to 61 Gy prior to surgical resection [10]. The Sonett study found 1, 2, and 5 year OS rates of 92.4%, 66.7%, and 46.2% respectively [10]. Although results from all these various studies are very promising, they are not confirmatory and call for further investigation.

Evaluation of failure patterns will help to justify the application of one treatment plan over another as well as indicate which options are resulting in failure. In order to answer that question this study examined failure patterns associated with stage IIIA and IIIB NSCLC. Specifically, a comparison was made between failure rates of definitive chemoradiation versus neoadjuvant concurrent chemoradiation followed by surgical resection. We hypothesized that given the poor local control rates with chemotherapy and radiation therapy alone, surgery would improve the outcomes but OS may not be improved given the mortality risk from surgery. These factors together could help to increase the currently dismal survival rate of this common cancer.

## Materials and Methods

At the University of Washington Medical Center (Seattle, WA) and the Puget Sound Veterans Administration (Seattle, WA), 836 patients were identified as being diagnosed with lung cancer between January 1994 and March 2007. Of these 92 were classified as having an initial diagnosis of stage IIIA or IIIB non-small cell lung cancer. These patients were analyzed retrospectively based on treatment plans with a focus on failure differences seen in concurrent chemoradiation alone (Group 1) versus neoadjuvant concurrent chemoradiation followed by surgical resection (Group 2). Patients were staged according to AJCC version 6 [12].

### Patient characteristics

Detailed characteristics of the 92 patients are outlined in Table 1. Group 1 (n = 76) contained 53 patients (69.7%) from UWMC and 23 (30.3%) from Puget Sound VA. The group broke down by gender with 53 (69.7%) male patients and 23 (30.3%) female patients. Patients ranged in age at time of diagnosis from 41 to 82 with a median age of 60. Of those patients in Group 2 (n = 16), 11 (68.75%) were from UWMC and 5 (31.25%) from Puget Sound VA. Also, 12 (75.0%) of the 16 were male and 4 (25.0%) were female. Patients ranged in age at time of diagnosis from 38 to 67 with a median of 60.

**Table 1:** Patient Characteristics

Characteristic	Group 1 (N = 76)	Group 2 (N = 16)
	Number (%)	Number (%)
Gender		
Male	53 (69.7)	12 (75.0)
Female	23 (30.3)	4 (25.0)
Median Age (range, years)	60 (41 - 82)	60 (38 - 67)
Histology		
Adenocarcinoma	10 (13.2)	1 (6.55)
Squamous	17 (22.4)	1 (6.25)
Large Cell	3 (3.9)	0
Unspecified NSCLC	46 (60.5)	14 (8.75)
Pretherapy Stage		
IIIA	26 (34.2)	13 (81.25)
IIIB	50 (65.8)	3 (18.75)

The zinc concentration is expressed as μg/dl. The results are shown as mean ± SEM.

Initially diagnosis classification was confirmed by pathology and CT reports. Specifically, Group 1 included 26 (34.2%) patients with pathological stage IIIA disease and 50 (65.8%) with stage IIIB disease. Group 2 included 13 (81.25%) patients identified as pathological stage IIIA disease and 3 (18.75%) as stage IIIB disease.

### Radiotherapy

Patients in Group 1 were planned to receive a definitive radiation dose to the primary tumor ranging from 57.6 to 70.0 Gy with a median of 61.0 Gy. The most common doses were 59.4 Gy (22.4%), 60.0 Gy (19.7%), and 61.0 Gy (17.1%). Only two patients did not complete their dosing due to toxicities (pneumonitis grade IV and esophagitis with unknown grade) from radiation treatment. The majority of those in Group 2 received an induction radiation dose to the primary tumor of 45 Gy (81.25%) prior to surgical resection, however, the range varied from 45.0 to 61.2 Gy with a median of 45.0 Gy. All patients in Group 2 completed their full treatment regimen.

### Chemotherapy

The majority of patients in Group 1 received a platinum combination chemotherapy treatment (88.2%) with 52 of the 76 patients (68.4%) treated with of a platinum plus etoposide and 15 of the 76 (19.7%) treated with platinum plus paclitaxel. Three other patients received single chemotherapy agents (platinum, paclitaxel, or etoposide) and the remaining six had unknown regimens. In comparison, 14 of the 16 patients in Group 2 received a chemotherapy regimen consisting of a platinum and etoposide. Two other patients in Group 2 received a combination of a platinum and paclitaxel.

### Surgery

Of the 16 patients in Group 2, 14 underwent lobe resection and 2 had pneumonectomies. There were no operative or postoperative associated deaths. The majority of patients underwent surgery about 1 - 2 months after induction chemoradiation (81.3%). Overall though, patients waited between 1 to 21 months before surgery. Five of the patients with stage IIIA disease were down staged after surgery from their initial diagnosis. One patient was down staged to stage IB, two to stage IIB, one to stage IA, and one had no evidence of disease. Additionally, one patient with initial stage IIIB disease was down staged after surgery to stage IIIA and another had no evidence of disease.

Access (Microsoft, Redmond, WA) was used to create and store information in a database and statistical analysis was computed using SPSS (SPSS 12.0.2, Inc., Chicago, IL). The Kaplan-Meier method was used to calculate survival rates with 95% confidence intervals [13].

## Results

### Response to therapy

**Figure 1:**
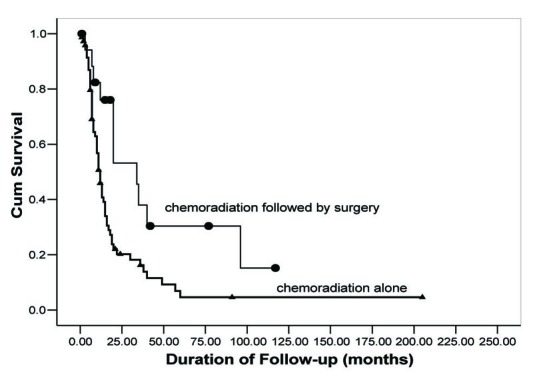
Overall survival as a function of treatment group. Comparison of OS rates in neoadjuvant chemoradiation followed by surgical resection (circles) versus concurrent chemoradiation alone (triangles).

OS rates were calculated using the Kaplan-Meier method and are shown in Figure 1. Follow-up for patients in Group 1 ranged from 15 days to 91 months with a median 10.5 months. In Group 2, follow-up for patients ranged from 7 to 117 months with a median of 20 months. OS for 2, 3, and 5 years was 19.7%, 15.7%, and 4.5%, respectively, in Group 1. In contrast, Group 2 demonstrated an improved OS (56.4%, 40.4%, 32.3%), (P = 0.003). Additionally, Group 2 demonstrated an improved median survival. Group 1 median survival was 11 months (95% CI: 8.8 - 13.2) versus 34 (95% CI: 8.7 - 59.3) in Group 2.

### Disease recurrence

**Figure 2:**
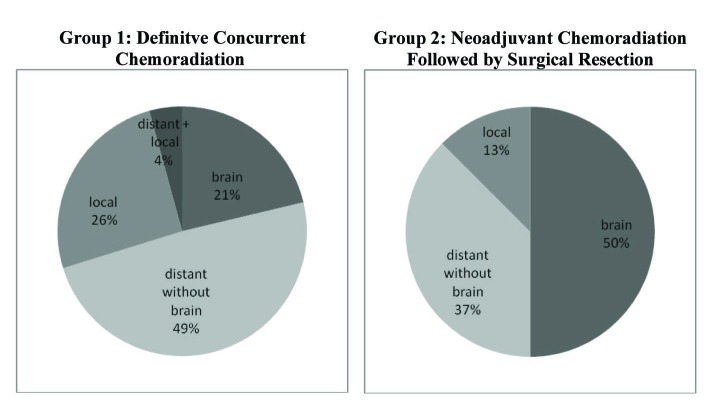
Rates of disease recurrence in both treatment groups. Group 1 had an overall recurrence rate of 61.8% (47 of 76) with the majority of recurrence occuring distantly without brain involvement. Group 2 showed a recurrence rate of 44.4% (8 of 18) with the majority occuring distantly in the brain. These recurrences are divided accordingly above.

Break down of pathological disease recurrence location is demonstrated in Figure 2. In Group 1, 47 of the 76 (61.8%) patients had pathologically documented recurrence as of last follow-up. Of the 47 patients with documented recurrence, 23 (48.9%) experienced distant recurrence without any brain involvement, 12 (25.5%) had only local recurrence, 10 (21.3%) had distant recurrence in the brain, and 2 (4.3%) had simultaneous presentation of both distant and local recurrence.

In contrast, 8 of the 16 patients (50.0%) in Group 2 had documented local or distant recurrence as of last follow-up. Of these 8, 4 (50%) had documented recurrence in the brain, 3 (37.5%) had distant recurrence without brain involvement, and 1 (12.5%) showed only local progression. From the recurrence data it appears that Group 1 had a greater incidence of distant recurrence without any brain involvement, whereas Group 2 had higher frequency of brain recurrence.

## Discussion

The OS rate of Group 2 (40.4% at three years) corroborates the findings from the most recent NSCLC Intergroup study 0139. Similarly to our study, the Intergroup study 0139 compared the OS rates of patients of those who received definitive concurrent chemoradiation treatment alone (Group 1) to those treated by induction concurrent chemoradiation followed by surgical resection (Group 2). Patients in both groups of the intergroup study received induction chemotherapy of cisplatin and etoposide along with a concurrent radiation dose to 45 Gy. Group 1 continued radiation treatment up to 61 Gy and Group 2 underwent surgical resection. The Intergroup study 0139 found results similar to the results seen in our study, with 3 year OS of 38.0% in Group 2 [7]. Our study, although, found median OS in this group to be longer than that found in the intergroup study (34.0 v 22.1 months). This finding does not necessarily discount the quality of either study, but rather demonstrates an improvement in long term survival that can be obtained with a trimodality approach to progressive non-small cell lung cancer.

Our retrospective study in contrast to the intergroup study however, had a lower 3 year survival in Group 1 (15.7% v 33.0%). The median survival seen in our study for this group was also reduced (11.0 v 21.7 months). The discrepancy in both OS and median survival for Group 1 most likely reflects both our inclusion of patients with more progressive disease (stage IIIB) and those of poor surgical candidacy. In contrast to our study, the intergroup study included only patients with stage IIIA disease whereas as the majority of our patients in Group 1 (50 of 76) had a pathological diagnosis of stage IIIB disease. The initial diagnosis of stage IIIB placed our patients in a category of more serious disease and worse initial prognosis compared to those patients with stage IIIA disease. This would then project that patients with stage IIIB disease would have a decreased overall and median survival compared to those with stage IIIA. Thus, further studies with an increased focus on stage IIIB disease is necessary to ultimately determine if the trimodality approach is both a feasible and effective treatment option for patients with stage IIIB disease.

The most recent update of the intergroup study continues to support the use of trimodality treatment [9]. In the 2005 update, Group 2 is shown to favor Group 1 with a 5 year progressive free survival and OS. Group 1 has a 5 year progressive free survival of 11.1% v 22.4% in Group 2. OS in Group 1 compared to Group 2 is 20.3% versus 27.2% respectively [7]. In comparison, our study found a slightly increased level of survival at 5 years for Group 2 at 32.3% but continued to have a lower survival for Group 1 with 4.5%. The decreased survival of Group 1 in our study most likely continues to reflect our inclusion for patients with the progressive stage IIIB disease. The 6 patients in this group with unknown chemotherapy regimens may have also contributed since it is possible they received substandard chemotherapy treatment. The difference in 5 year survival rate for Group 2 on the other hand, may be related to the larger size of the intergroup study with their inclusion of 194 patients compared to our 16.

A phase II study conducted by the Southwest Oncology Group also demonstrated an improvement in OS with concurrent induction chemoradiation followed by surgical resection in patients with both stage IIIA and stage IIIB [6]. Patients in this study underwent inductive chemotherapy of cisplatin and etoposide with a radiation dose to 45 Gy. In the study, 75 patients (60%) with stage IIIA disease were enrolled in the study with 57 undergoing resection. The stage IIIB disease group contained 51 patients (40%) and 32 underwent surgical resection. This study found 2 and 3 year OS in trimodality treated patients with stage IIIA to be 37% and 27% respectively [5]. In those with stage IIIB disease, 2 and 3 year OS was 39% and 24% respectively [5]. The 2 and 3 year OS in Group 2 of our study (56.4%, 40.4%) then shows an improvement to this study. Our findings should be compared primarily to the patients with stage IIIA of this study since, of our 16 patients, only 3 demonstrated stage IIIB disease. Despite this, our study demonstrates promising results and the improved 2 and 3 OS in our study continues to support a trimodality based approach to patients with progressive non-small cell lung cancer.

The European Organization for Research and Treatment of Cancer-Lung Cancer Group also conducted a study comparing the survival of patients who underwent neoadjuvant chemotherapy followed by radiotherapy versus surgical resection in patients with stage IIIA disease [11]. The study found the median and 5 year OS to be similar in both the radiotherapy and surgical groups (16.4 v 17.5 months and 15.7% v 14% respectively) [11]. The decreased 5 year OS in this study compared to our own (32.3%) further supports the benefit of a trimodality approach for patients who are capable of undergoing surgical resection.

Lastly, a study conducted by the University of Maryland School of Medicine shows an improved OS rate for trimodality treatment when compared to our study. In the Maryland study 40 patients with non-small cell lung cancer received platinum chemotherapy treatment with concurrent radiation to 59 Gy followed by surgical resection [14]. The Maryland study found OS rates at 2 and 5 years of 66.7% and 46.2% respectively with a median OS of 53 months [14]. The improved OS seen in the Maryland study compared to our study most likely reflects the inclusion of patients with less severe disease (IIB), a higher dose of radiation therapy, and a higher performance status.

Disease recurrence rates were also calculated in the Maryland study. They showed decrease in recurrence when compared to our study of 35% v 47.1% respectively. In our study it was difficult to draw strict conclusions concerning the prevelance and location of disease recurrence between the two groups since Group 2 had so few patients overall with recurrence. Even one additional patient in Group 2 with recurrence would have significantly altered the recurrence distribution for that group.

The OS of patients in our study receiving trimodality treatment demonstrates increased survival rates and promising outcomes compared with those receiving concurrent chemoradiation alone, as well as validating former studies. The study then suggests that patients with stage IIIA and, perhaps, IIIB non-small cell lung cancer and a high performance status should be considered for trimodality treatment. Due to the small sample size and retrospective nature of this study, investigation of trimodality treatment for locally advanced non-small cell lung cancer should continue. A large scale prospective study would be necessary to ultimately confirm the advantage of this treatment option to increase both overall and progressive free survival. A new standard of care could then be defined.
